# ALS plasma biomarkers reveal neurofilament and pTau correlate with disease onset and progression

**DOI:** 10.1002/acn3.70001

**Published:** 2025-02-06

**Authors:** Eleanor V. Thomas, Changee Han, Woo Jae Kim, Seneshaw Asress, Yingjie Li, Jennifer A. Taylor, Marla Gearing, Christina N. Fournier, Zachary T. McEachin, Nicholas T. Seyfried, Jonathan D. Glass

**Affiliations:** ^1^ Department of Neurology Emory University Atlanta Georgia 30322 USA; ^2^ Center for Neurodegenerative Diseases Emory University Atlanta Georgia 30322 USA; ^3^ Department of Human Genetics Emory University Atlanta Georgia 30322 USA; ^4^ University of Chicago Chicago Illinois 60637 USA; ^5^ Department of Cell Biology Emory University Atlanta Georgia 30322 USA; ^6^ Laboratory of Translational Cell Biology Emory University Atlanta Georgia 30322 USA; ^7^ Department of Biochemistry Emory University Atlanta Georgia 30322 USA

## Abstract

**Objective:**

We performed a pilot screen to assess the utility of the NULISA™ (Nucleic‐acid‐Linked Immuno‐Sandwich Assay) platform in the identification of amyotrophic lateral sclerosis (ALS) biomarkers.

**Methods:**

Plasma from 86 individuals (48 ALS, 18 asymptomatic C9*orf*72 repeat expansion carriers (AsymC9), and 20 healthy controls) was analyzed via a multiplexed NULISA™ assay that includes 120 neurodegeneration‐associated proteins. Statistical analysis of NULISA™ results was performed to identify proteins differentially expressed in plasma and their correlation with disease‐associated parameters.

**Results:**

ALS plasma showed elevation of the established biomarkers, neurofilament light chain (NEFL) and neurofilament heavy chain (NEFH). Compared to controls and AsymC9, microtubule‐associated protein tau (MAPT), phosphorylated tau 181 (pTau181), phosphorylated tau 217 (pTau217), phosphorylated tau 231 (pTau231), and phosphorylated TDP‐43 (pTDP‐43) were elevated in ALS. NEFL levels positively correlated with pTau181, pTau217, pTau231, and pTDP‐43. MAPT and pTDP‐43 were also correlated with pTau181, pTau217 and pTau231. Elevated pTau was negatively correlated with survival and ALSFRS‐R. Spinal onset ALS was associated with higher pTau181, pTau217, and pTau231.

**Interpretation:**

We confirm previous reports showing elevated pTau181 in ALS plasma and show elevation of other phosphorylated tau forms, pTau217 and pTau231, typically observed in Alzheimer's disease. We provide preliminary data showing the detection and elevation of pTDP‐43‐409/410 in a subset of ALS samples compared to healthy controls. Neurofilament and tau levels are highly correlated suggesting their elevation may reflect a common pathology and disease state. Total and phosphorylated tau are correlated with multiple disease measures, such as ALS duration, ALSFRS‐R, and site of onset.

## Introduction

Plasma‐based biomarkers for neurodegenerative diseases, such as ALS, show great promise both in the clinical and research settings. Neurofilament, one of the first identified and most robust, correlates with the rate of axon loss and disease progression and prognosticates survival.^1^, ^2^, ^3^, ^4^, ^5^ Neurofilament light chain (NEFL) can be used to detect risk for ALS disease conversion in at‐risk individuals carrying ALS‐causing mutations, such as SOD1 pathogenic variants^6^ and is elevated in ALS due to C9*orf*72 repeat expansions.^7^ Lowering of neurofilament in plasma has been used in clinical trials as a surrogate for survival and was a factor in the FDA approval of Tofersen for patients carrying SOD1 pathogenic variants.^8^ In addition to NEFL, there is a great need for the identification of new biomarkers that may identify different stages of the disease, describe novel pathologies, and that may be used in combination with NEFL.^9^

Elevation of plasma phosphorylated Tau181 (pTau181), phosphorylated Tau 217 (pTau217) and phosphorylated Tau (pTau231) are proposed as biomarkers of Alzheimer's Disease pathology,^10^ and recently pTau181 was also discovered to be elevated in ALS plasma.^11^, ^12^ Although pTau181 has also been detected in ALS CSF, it was not elevated to the same extent as plasma.^13^, ^14^ For example, in one study the phosphorylated tau to total ratio was decreased in ALS CSF relative to healthy seniors,^14^ and in another study pTau181 in CSF was not correlated with pTau181 in plasma,^15^ Additionally, mass‐spectrometry based proteomic methods show that total tau (MAPT) is not elevated in CSF from ALS patients.^16^ Given this discordance between the elevation of tau in the plasma in ALS and relatively little difference or a decrease in CSF, tau elevation in ALS plasma has been hypothesized to originate from lower motor neuron degeneration. One study hypothesized a peripheral source for pTau181 and showed it was most correlated with lower motor neuron impairment.^15^

Tar DNA binding protein 43 (TDP‐43) is a protein of special relevance to ALS, and when phosphorylated, is excluded from the nucleus and forms cytoplasmic aggregates.^17^ This finding is shared in both C9ALS and sporadic ALS and is a pathological hallmark of the disease. Different approaches have been used to detect TDP‐43 in ALS blood, including ELISA‐based methods detecting total and phosphorylated TDP‐43 in plasma^18^ and total TDP‐43 in platelets.^19^ These studies showed modest differences in total and phosphorylated TDP‐43 (pTDP‐43) between ALS and healthy controls. Another recent approach isolated plasma small extracellular vesicles (sEVs) and showed elevated sEV 3R/4R tau ratio and total TDP‐43 in ALS.^20^ Nevertheless, robust detection of pTDP‐43 in small volumes of ALS patient plasma remains difficult and is an active area of interest, as it has the potential to provide a plasma‐based measurement for TDP‐43 proteinopathy in ALS patients and to inform when TDP‐43 aggregation starts in pre‐clinical asymptomatic C9 expansion carriers.

In this study, we profiled plasma from individuals with ALS, asymptomatic C9 repeat expansion carriers (AsymC9), and healthy controls, to determine whether this novel, ultra‐sensitive immunoassay will both detect known ALS plasma biomarkers, as well as reveal new targets of interest. The Nucleic‐acid‐Linked Immuno‐Sandwich Assay (NULISA™) CNS platform employs oligonucleotide‐conjugated antibodies to amplify the signal from 122 neurodegeneration‐associated proteins.^21^ Here, we detect the elevation of the known ALS biomarkers, neurofilament, and pTau181, as well as additional pTau species and novel targets of interest.

## Methods

### Study population and sample collection

We performed a pilot study to test the NULISASeq™ platform in a cohort of 50 ALS patients, 18 asymptomatic C9 carriers, and 20 controls. ALS cases were diagnosed and treated at the Emory ALS Center from 2007 to 2023. Emory neurologists cared for these patients throughout their disease course and diagnoses were made using established clinical criteria. A database is maintained for all clinical samples allowing deep clinical phenotyping, such as sex, date and age of onset, date of death, disease duration, and ALSFRS‐R.^22^ All participants who provided plasma samples were screened for C9*orf*72 repeat expansions and SOD1 pathogenic variants. The study cohort was enriched for genetic causes of ALS and contained 20 ALS cases with C9*orf*72 expansions and 10 cases with SOD1 pathogenic variants. Within the ALS group two patients with exceptionally long disease durations >9 years, were determined to be outliers, and eliminated from statistical analysis. Plasma samples were acquired under the proper Institutional Review Board (IRB) protocol with informed consent from the research subjects. All experiments conformed to the principles set by the WMA Declaration of Helsinki and the Department of Health and Human Services Belmont Report.

### NULISA platform

Plasma was thawed and centrifuged at 10,000 g for 10 minutes, and the supernatant was analyzed via a multiplexed NULISA™ assay that includes 120 neurodegeneration‐associated proteins. Briefly, in this panel, a capture antibody conjugated to a partially double‐stranded DNA molecule containing a poly‐A tail and a detection antibody with another partially double‐stranded DNA molecule and a biotin group are incubated with patient plasma. These antibodies bind the target of interest forming immunocomplexes that are captured with oligo‐dT beads. Following washing and removal of oligo‐dT beads and subsequent pull‐down of the detection antibody with streptavidin, Next Generation Sequencing (NGS) of the DNA reporter is used to measure targets of interest. Detailed methods are outlined in Feng, et al.^21^ NGS detection of protein levels are reported as NULISA Protein Quantified (NPQ).

### Data normalization and quality control

Normalization of target NPQ counts is performed by dividing those counts by internal controls in each sample well. Inter‐plate normalization is performed. NPQ counts are then rescaled and log‐2 transformed. Therefore, differences in NPQ reflect log2‐fold change. The plate‐specific limit of detection (LOD) is also calculated for each analyte by calculating the mean plus 3 standard deviations for the negative control, which is a blank sample. The LOD is then rescaled and log2‐transformed as for NPQ. Sample controls and coefficient of variation are calculated from pooled plasma sample replicates on each plate.

### Statistical analysis

Statistical analysis of NULISA results was performed using a one‐way ANOVA with Tukey's Honest Significant Difference (HSD) post‐hoc correction for multiple groups to identify proteins that were differentially expressed in plasma. This analysis was done using R. Boxplots were generated in Python. Correlation with disease‐associated parameters, such as disease duration and ALSFRS‐R, was calculated with Spearman's coefficient and analyzed in GraphPad Prism. Comparison of neurofilament and tau levels between bulbar and spinal onset was performed via student's t‐test and plotted in GraphPad Prism.

## Results

### Detection of elevated phosphorylated tau and pTDP‐43 in ALS plasma

We profiled plasma from 48 people with ALS, 18 asymptomatic C9 repeat expansion carriers (AsymC9), and 20 healthy controls. Demographic data from this study population are shown in Table 1. There were no significant differences in age across controls, AsymC9, and ALS cases. A high proportion of familial ALS cases was included in this study. Of the 122 targets included on the NULISA CNS panel, there were statistically significant differences (*p* > 0.05) in 23 neurodegeneration‐associated proteins (Table S1).

**Table 1:** Sample characteristics.

	Control (*n* = 20)	Asym C9 (*n* = 18)	ALS (*n* = 48)
Age at plasma (years)	58.3 ± 7.9	57.2 ± 17.7	57.7 ± 10.9
Age at death (years)	–	–	59.1 ± 11.0
Disease duration (mos.)	–	–	28.0 ± 22.0
C9ORF72 (%)	–	–	20 (41.7%)
SOD1 (%)	–	–	9 (18.8%)
Gender (%)			
Female	16 (80%)	12 (66.7%)	25 (52.1%)
Male	4 (20%)	6 (33.3%)	23 (47.9%)
ALS region of onset (%)			
Bulbar			14 (29.2%)
Spinal			34 (70.8%)
ALSFRS‐R			33.6 ± 9.2

**Figure 1:**
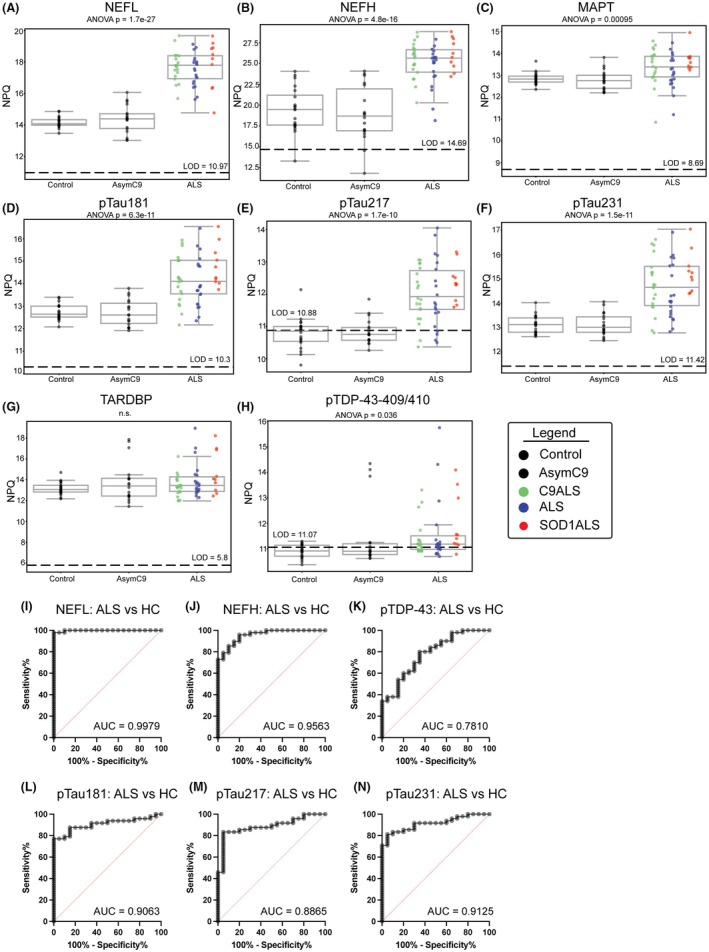
Elevation of phosphorylated Tau and TDP‐43 in ALS plasma. NULISA Protein Quantification (NPQ) for each protein is shown on the y‐axis and the limit of detection (LOD) is shown with a dotted line. NPQ for Control, asymptomatic C9, and ALS are compared for (A) NEFL (ANOVA *p* = 2.0e^−25^), (B) NEFH (ANOVA *p* = 2.9e^−25^), (C) MAPT (ANOVA *p* = 0.01), (D) pTau181 (ANOVA *p* = 1.9e^−9^), (E) pTau217 (ANOVA *p* = 4.3e^−9^), (F) pTau231 (ANOVA *p* = 6.1e^−10^), (G) TARDP (ANOVA *p* = 0.29, n.s.) and (H) pTDP‐43 (ANOVA *p* = 0.036). ROC Curve analysis comparing ALS to healthy controls. (I) NEFL AUC = 0.9979, 95% confidence interval 0.9923–1.000, (J) NEFH AUC = 0.9563, 95% confidence interval [0.9128–0.9997], (K) pTDP‐43 AUC = 0.7810, 95% confidence interval [0.6643–0.8977], (L) pTau181 AUC = 0.9063, 95% confidence interval [0.8357–0.9768, (M) pTau217 AUC = 0.8865, 95% confidence interval [0.8053–0.9676], (N) pTau231 AUC = 0.9125, 95% confidence interval [0.8465–0.9785].

The established ALS biomarkers, neurofilament light and heavy chains (NEFL and NEFH), were robustly elevated in ALS plasma (ANOVA *p* = 2.0e‐25; ANOVA *p* = 2.9e‐25, respectively) showing that this approach reliably detects known ALS‐associated biomarkers. pTau181, pTau217 and pTau231 were also robustly elevated in ALS plasma (pTau181 ANOVA *p* = 1.9e‐9; pTau217 ANOVA *p* = 4.3e‐9; pTau231 ANOVA *p* = 6.1e‐10). Note that phosphorylated tau was elevated across all genetic subgroups, C9ALS, SOD1ALS, and sporadic ALS and was not elevated in AsymC9. Total TDP‐43 (TARDBP) was reliably detected by NULISASeq™ in ALS, AsymC9, and control samples, but it was not significantly different in this small cohort. Notably, phosphorylated TDP‐43 (pTPD‐43‐409/410) was differentially abundant (ANOVA *p* = 0.036) and was elevated in ALS. While the majority of AsymC9 cases did not have elevated pTDP‐43, three samples had pTDP‐43 levels as high as those measured in ALS cases (Fig. 1). None of the Asymptomatic C9 research participants who provided these samples had yet developed ALS or frontotemporal dementia at the time of this analysis, and neurofilament levels in these 3 participants were comparable to normal controls (Fig. S1).

Receiver operating curve (ROC) analysis showed high discrimination between ALS and controls using NEFL (AUC 0.9979, 95% confidence interval CI [0.9923–1.000]), NEFH (AUC 0.9563, CI [0.9128–0.9997]), pTau181 (AUC 0.9064, CI [0.8357–0.9768]), pTau217 (AUC 0.8865, CI [0.8053–0.9676]), and pTau231 (AUC 0.9125, CI [0.8465–0.9785]). NEFL and NEFH showed the highest diagnostic accuracy, but pTau181, pTau217, and pTau231 were also highly accurate. (Fig. 1).

### Correlation of tau and neurofilament with pTDP‐43 and clinical parameters

**Figure 2:**
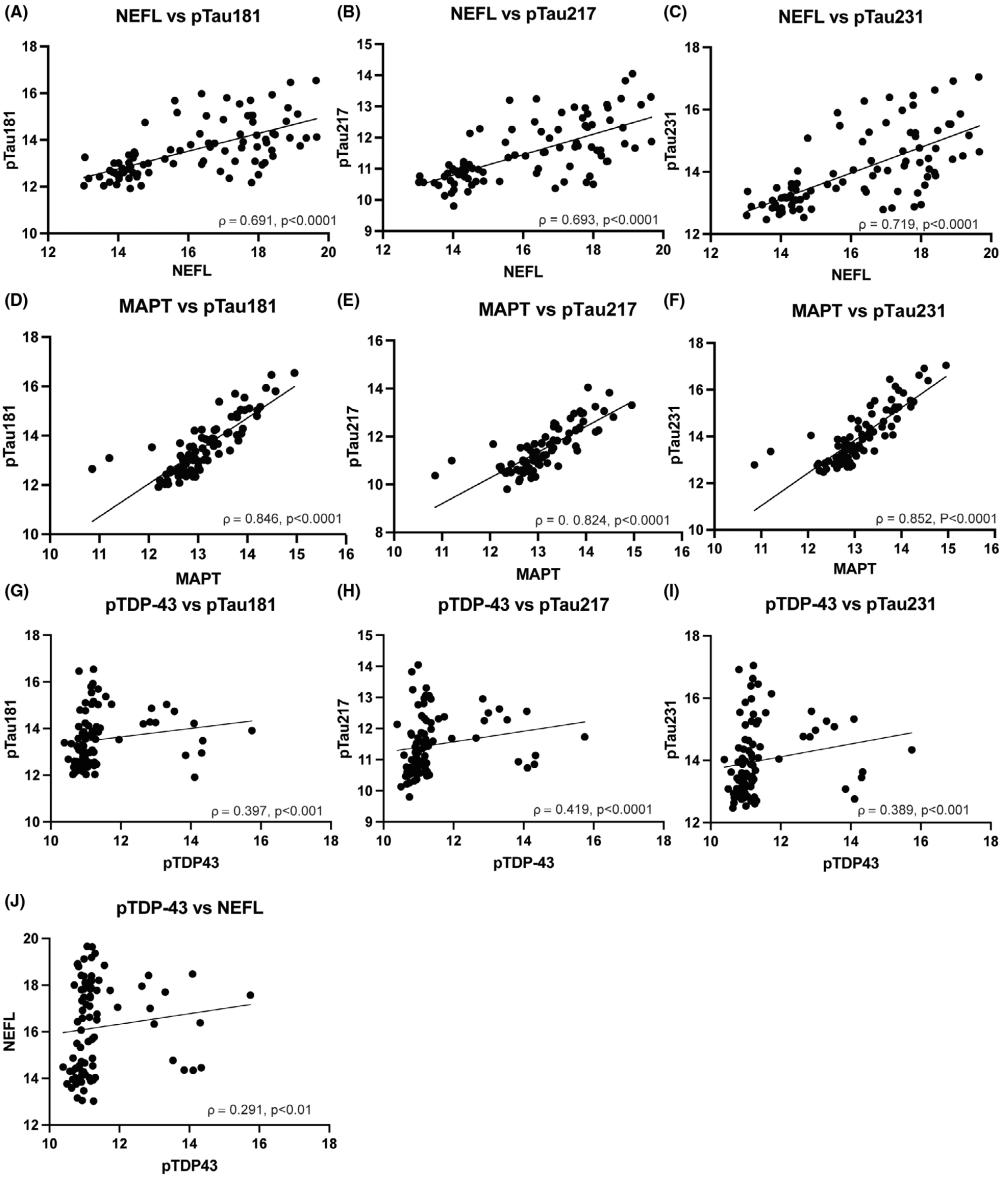
Phosphorylated Tau, Neurofilament and pTDP‐43 are correlated. Scatter plot of NEFL vs phosphorylated tau (A–C) and MAPT vs phosphorylated tau (D–F) and pTDP‐43 vs phosphorylated tau and NEFL (G–J) shown with linear regression including all samples (Controls, Asym C9 and ALS). NEFL is positively correlated with pTau181 (*ρ* = 0.691, *p* < 0.0001), pTau217 (*ρ* = 0.693, *p* < 0.0001), and pTau231 (*ρ* = 0.719, *p* < 0.0001). MAPT is positively correlated with pTau181 (*ρ* = 0.846, *p* < 0.0001), pTau217 (*ρ* = 0.824, *p* < 0.0001), and pTau231 (*ρ* = 0.852, *p* < 0.0001). pTDP‐43 is positively correlated with pTau181 (*ρ* = 0.397, *p* < 0.001), pTau217 ρ = 0.419, *p* < 0.0001), pTau231 (*ρ* = 0.389, *p* < 0.001), and NEFL (*ρ* = 0.291, *p* < 0.01).

NEFL was correlated with pTau181 (*r* = 0.69, *p* < 0.0001), pTau217 (*r* = 0.69, *p* < 0.0001), and pTau231(*r* = 0.72, *p* < 0.0001). Similarly, MAPT was highly correlated with pTau181 (*r* = 0.846, *p* < 0.0001), pTau217 (*r* = 0.824, *p* < 0.0001) and pTau231 (*r* = 0.852, *p* < 0.0001) as shown in Figure 2. There was a weak positive correlation between pTDP‐43 and all pTau species (pTau181 *r* = 0.397, *p* < 0.001; pTau217 *r* = 0.419, *p* < 0.0001, pTau231 *r* = 0.389, *p* < 0.001) as well as NEFL (*r* = 0.291, *p* < 0.01).

**Figure 3:**
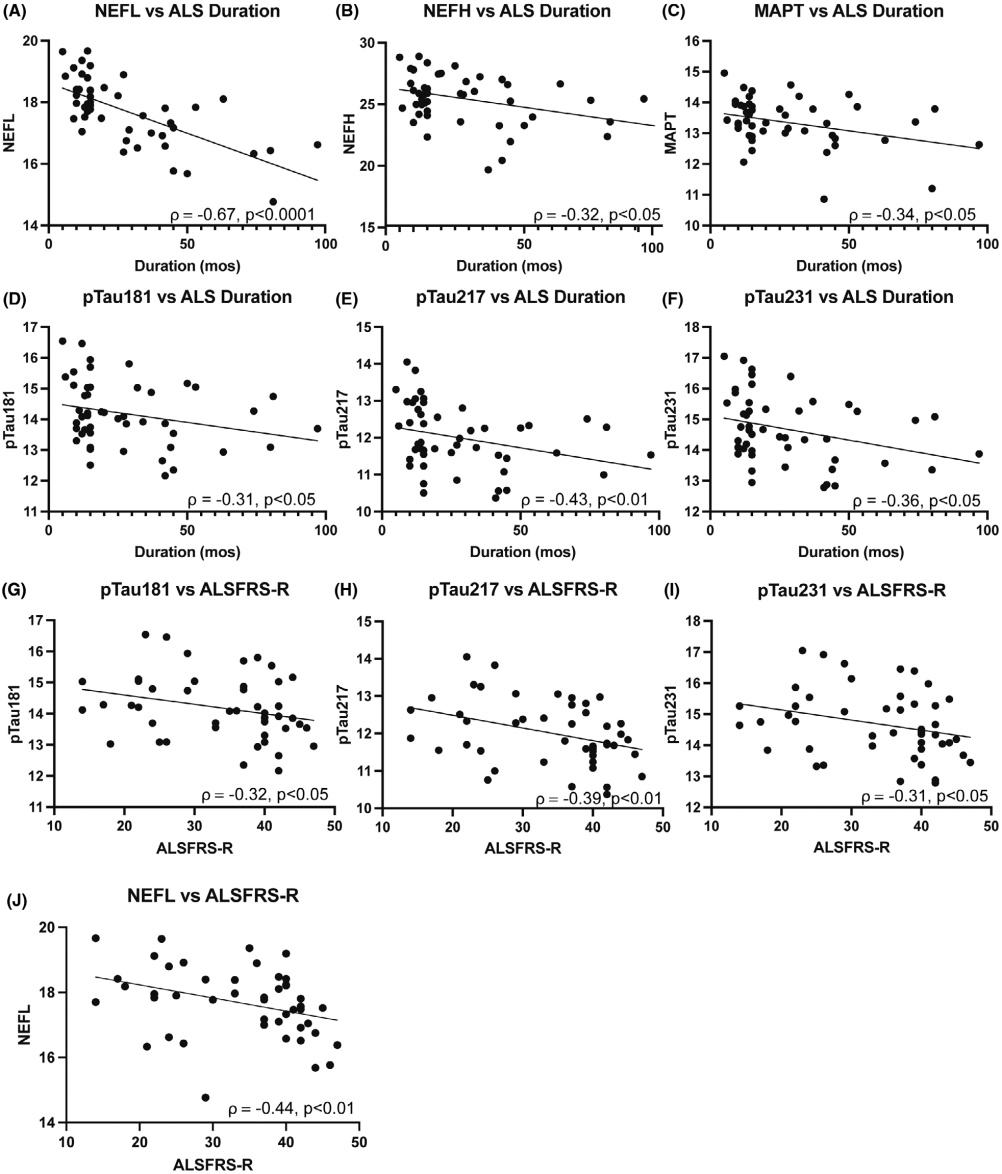
Elevated phosphorylated Tau correlates with disease severity. NEFL, MAPT, pTau181, pTau217, and pTau231 are negatively correlated with ALS disease duration (A–F). ALSFRS‐R was available in 46/48 ALS cases. pTau181, pTau217, pTau231, and NEFL are negatively correlated with ALSFRS‐R (G–J).

Next, we tested whether pTau correlates with disease‐associated parameters. ALS duration and pTau181, pTau217, and pTau231 were negatively correlated suggesting that elevated phosphorylated tau may be associated with more aggressive disease (Fig. 3). ALSFRS‐R within 1 month of plasma sampling was available for 46/48 ALS cases, and total ALSFRS‐R score was negatively correlated with pTau181, pTau217, pTau231, and NEFL. There was a trend towards higher pTau181, pTau217 and pTau231 levels in older individuals in both control and ALS groups, but this did not reach significance except for pTau217 in ALS (Fig. S2). Note that NEFL has been shown to increase with normal aging.^23^

### Elevation of tau in spinal onset ALS

**Figure 4:**
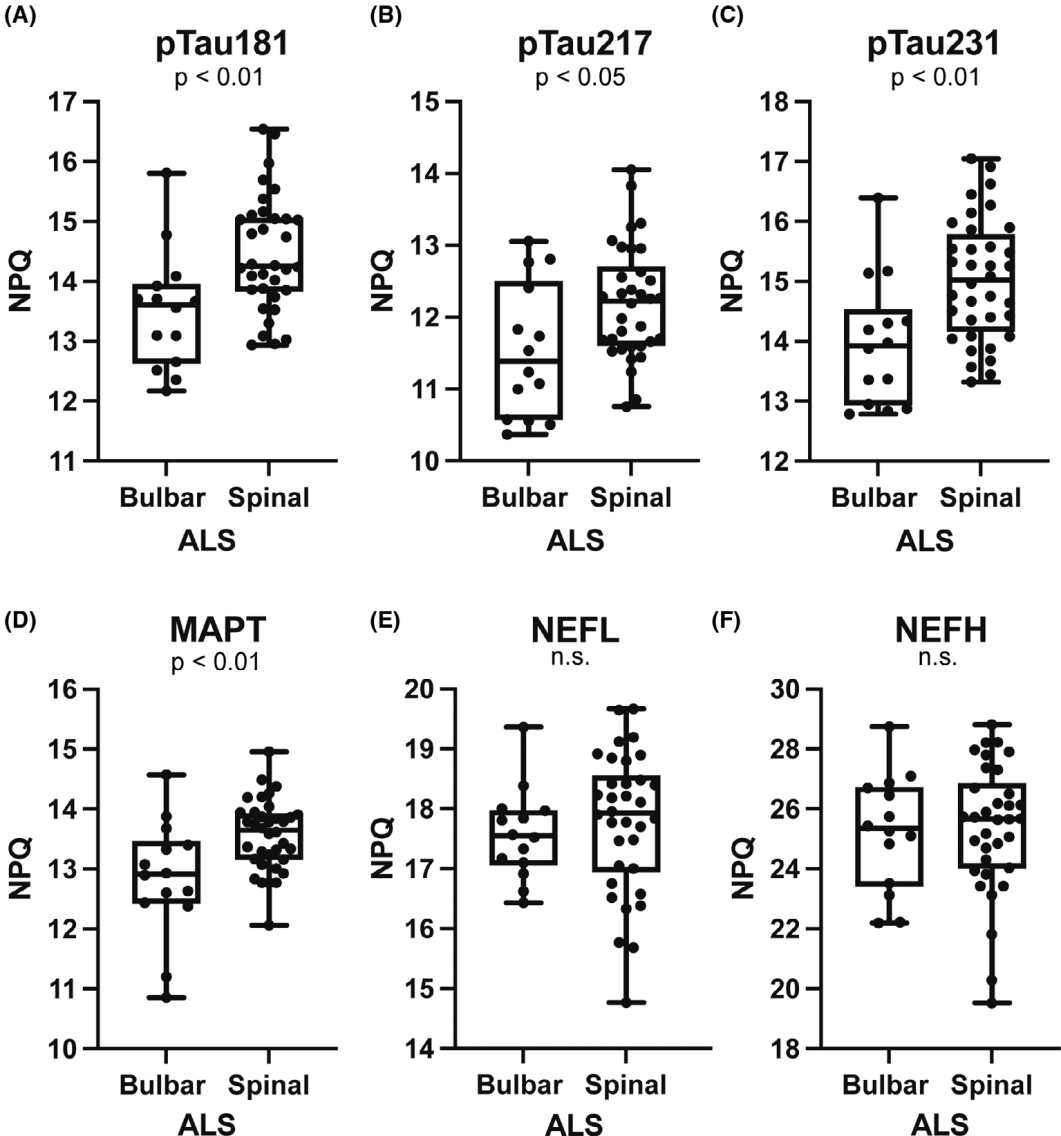
Phosphorylated and total tau are higher in spinal onset ALS. Spinal onset (*n* = 34) and bulbar onset (*n* = 14) ALS were compared by Student's t‐test. (A–D) pTau181, pTau217, pTau231 and MAPT were significantly higher in spinal onset ALS cases than bulbar onset ALS cases (pTau181 *p*‐value = 0.003; pTau217 *p*‐value = 0.016; pTau231 *p*‐value = 0.002; MAPT *p*‐value = 0.0025). There was no difference in NEFH and NEFL between spinal and bulbar onset cases (E and F).

ALS presents with phenotypic variability often categorized by site of onset, and we asked whether levels of pTau were associated with spinal vs. bulbar onset disease. Patients with spinal onset ALS showed greater elevation of pTau181, pTau217, pTau231, and MAPT as compared to bulbar onset disease, agreeing with previous data showing higher pTau181 in spinal onset ALS.^12^ There was no difference between NEFL and NEFH in spinal and bulbar onset ALS in this cohort (Fig. 4).

## Discussion

This study builds upon a large body of evidence showing that the elevation of phosphorylated tau is not unique to Alzheimer's Disease, as pTau181 has previously been shown to be elevated in ALS. In addition, we found that two other phosphorylated tau species, pTau217 and pTau231, are also elevated in ALS plasma. Elevation of pTau and NEFL may reflect aspects of a shared pathology given their high correlation with one another. Previous studies speculated that higher pTau181 in plasma may be secondary to peripheral nerve degeneration in ALS,^12^ and this is also supported by our data showing that all tau species show a greater degree of elevation in spinal onset ALS plasma.

The presence of various pTau species, notably Thr175, have been shown in ALS cortex and spinal cord tissue sections,^24^, ^25^, ^26^ but these studies also demonstrated that pTau was present in some controls.^25^ In most cases, these neuropathological analyses were performed with different antibodies (AT8, PHF‐1) against different phosphorylation specific epitopes, so it is difficult to determine whether NULISA is measuring the same pTau species that are found in neuropathological tangles or pre‐tangles. Further quantitative and morphological studies against pTau181 and pTau217, may further clarify cell populations responsible for pTau elevations in plasma.

Of the proteins in the NULISA™ CNS panel, NEFL was the most sensitive in discriminating ALS from controls. However, all pTau species showed high accuracy in discriminating ALS cases from controls. Measurement of total TDP‐43 and pTDP‐43 in plasma have been challenging and limited by the sensitivity of ELISA‐based assays, variability of TDP‐43 and pTDP‐43 levels within control and ALS/FTLD populations, and the insolubility of TDP‐43 aggregates in biofluids.^27^ However, quantitative measurement of TDP‐43 and pTDP‐43 in plasma was possible with NULISA™, and a significant difference was noted for pTDP‐43. Somewhat surprisingly, however, we also found elevated levels of pTDP43 in select SOD1ALS patients. Though pTDP‐43 has been reported in SOD1ALS,^28^ this finding contrasts with the majority of data from post‐mortem studies demonstrating a lack of pTDP43 cytoplasmic inclusions in patients carrying SOD1 pathogenic variants.^29^ Further studies will be needed to confirm pTDP‐43 elevation in SOD1ALS plasma, and if so, to determine its cellular source.

It was possible to detect elevated pTDP‐43 in plasma of three asymptomatic C9 expansion carriers. This finding requires further validation in larger cohorts; however, it suggests that pTDP‐43 pathology may precede disease by many years. Further longitudinal measurements of pTDP‐43 in C9 expansion carriers may reveal the kinetics of pTDP‐43 elevation and its clinical relevance towards disease onset and progression. Interestingly, recent studies have demonstrated aggregation of TDP‐43 in non‐CNS tissues, such as colon, skin, and lymph node, many years prior to the development of ALS.^30^

The limitations of this pilot study include the relatively small number of patients and controls used, and the inclusion of larger sample sizes will be important in replicating these findings. Nevertheless, the sensitivity of this assay allowed for the measurement of statistically robust differences in this small cohort. Neurodegeneration controls, such as Alzheimer's patients, were not available at the time of this analysis, but it will also be important to include other neurodegenerative diseases in future studies to determine how pTau levels compare. The ALS study population was enriched for genetic forms, and for this reason, may be somewhat less representative of a general ALS population. Given the presence of gene therapies for SOD1ALS and in development for C9ALS, the establishment of clinical and pre‐clinical biomarkers is especially relevant in these genetic subgroups. Despite this enrichment for genetic forms of ALS, levels of neurofilament, pTau, and pTDP‐43 were similar across all ALS subgroups and findings appeared generalizable to sporadic ALS.

Certain analytes, such as NEFL, MAPT, pTau181, pTau231, MAPT, and total TDP‐43 (TARDBP), were consistently above the limit of detection of the NULISASeq™ assay. These were even robustly measured in all control samples suggesting that there is a normal steady‐state amount of these neuronal proteins that is “leaked” into plasma. In contrast, pTau217 and pTDP‐43‐409/410 measurements were below the LOD in many samples, in particular, controls. In AD pTau217 has been proposed as a biomarker with particularly high accuracy in discriminating disease.^31^Similarly, pTDP‐43 is a pathological hallmark of disease in ALS and is rare in control neuropathological specimens.^32^ Therefore, the very low levels of these analytes in control samples, below what can be reliably measured by NULISASeq™, likely reflect their true paucity in non‐disease states. As the LOD is defined as three standard deviations above the mean of blank test samples, this is a strict threshold, and some true measurements are likely below this threshold. Further validation in larger cohorts will better help determine reference ranges for real‐world plasma samples from diverse control, healthy aged, and neurodegeneration populations.

Future studies may also use this platform to obtain longitudinal measurements of NEFL, pTau, and pTDP‐43, which may reveal different disease stages. It will be important to determine the relative kinetics of NEFL, pTau and pTDP‐43 elevation and understand how these proteins vary in a particular patient over time. Longitudinal measurements in asymptomatic C9 carriers will be particularly interesting as it may be possible to track the development of TDP‐43 pathology and identify triggers of TDP‐43 proteinopathy. Future neuropathological studies may also ascertain which neuronal populations are most responsible for elevated pTau and pTDP‐43, and whether central or peripheral Tau is being measured in blood. This study shows the potential for sensitive multiplexed protein arrays in measuring ALS‐associated proteins in plasma, associating these markers for disease with clinical parameters, and identifying and monitoring pre‐clinical pathologies.

## Author Contributions

JDG and NTS conceived the project. EVT analyzed and interpreted data. CH and WJK provided informatics support. CNF, EVT, JAT, and JDG collected clinical data and plasma samples. SA and YL provided technical support. MG provided conceptual and technical expertise. NTS and JDG oversaw project administration and funding acquisition. EVT wrote the initial manuscript draft. EVT, CNF, ZTM, NTS, and JDG revised and edited the manuscript.

## Conflict of Interest

There are no conflicts of interest to report.

## Supporting information


Figure S1.

Figure S2.

Table S1.

**Caption S1**.

## Data Availability

Data files are available from authors on request.

## References

[1] Boylan K , Yang C , Crook J , et al. Immunoreactivity of the phosphorylated axonal neurofilament H subunit (pNF‐H) in blood of ALS model rodents and ALS patients: evaluation of blood pNF‐H as a potential ALS biomarker. J Neurochem. 2009;111:1182‐1191. 19765193 10.1111/j.1471-4159.2009.06386.x

[2] Brettschneider J , Petzold A , Sussmuth SD , Ludolph AC , Tumani H . Axonal damage markers in cerebrospinal fluid are increased in ALS. Neurology. 2006;66:852‐856. 16567701 10.1212/01.wnl.0000203120.85850.54

[3] Li S , Ren Y , Zhu W , Yang F , Zhang X , Huang X . Phosphorylated neurofilament heavy chain levels in paired plasma and CSF of amyotrophic lateral sclerosis. J Neurol Sci. 2016;367:269‐274. 27423602 10.1016/j.jns.2016.05.062

[4] Steinacker P , Feneberg E , Weishaupt J , et al. Neurofilaments in the diagnosis of motoneuron diseases: a prospective study on 455 patients. J Neurol Neurosurg Psychiatry. 2016;87:12‐20. 26296871 10.1136/jnnp-2015-311387

[5] Thouvenot E , Demattei C , Lehmann S , et al. Serum neurofilament light chain at time of diagnosis is an independent prognostic factor of survival in amyotrophic lateral sclerosis. Eur J Neurol. 2020;27:251‐257. 31437330 10.1111/ene.14063

[6] Benatar M , Wuu J , Andersen PM , Lombardi V , Malaspina A . Neurofilament light: a candidate biomarker of presymptomatic amyotrophic lateral sclerosis and phenoconversion. Ann Neurol. 2018;84:130‐139. 30014505 10.1002/ana.25276 PMC11348288

[7] Gendron TF , Group CONS , Daughrity LM , et al. Phosphorylated neurofilament heavy chain: a biomarker of survival for C9ORF72‐associated amyotrophic lateral sclerosis. Ann Neurol. 2017;82:139‐146. 28628244 10.1002/ana.24980 PMC5676468

[8] Miller TM , Cudkowicz ME , Genge A , et al. Trial of antisense oligonucleotide Tofersen for SOD1 ALS. N Engl J Med. 2022;387:1099‐1110. 36129998 10.1056/NEJMoa2204705

[9] Chen X , Chen Y , Wei Q , et al. Assessment of a multiple biomarker panel for diagnosis of amyotrophic lateral sclerosis. BMC Neurol. 2016;16:173. 27634542 10.1186/s12883-016-0689-x PMC5024522

[10] Gonzalez‐Ortiz F , Kac PR , Brum WS , Zetterberg H , Blennow K , Karikari TK . Plasma phospho‐tau in Alzheimer's disease: towards diagnostic and therapeutic trial applications. Mol Neurodegener. 2023;18:18. 36927491 10.1186/s13024-023-00605-8 PMC10022272

[11] Cousins KAQ , Shaw LM , Shellikeri S , et al. Elevated plasma phosphorylated tau 181 in amyotrophic lateral sclerosis. Ann Neurol. 2022;92:807‐818. 35877814 10.1002/ana.26462 PMC9588516

[12] Vacchiano V , Mastrangelo A , Zenesini C , et al. Elevated plasma p‐tau181 levels unrelated to Alzheimer's disease pathology in amyotrophic lateral sclerosis. J Neurol Neurosurg Psychiatry. 2023;94:428‐435. 37012065 10.1136/jnnp-2022-330709

[13] Scarafino A , D'Errico E , Introna A , et al. Diagnostic and prognostic power of CSF tau in amyotrophic lateral sclerosis. J Neurol. 2018;265:2353‐2362. 30116940 10.1007/s00415-018-9008-3

[14] Grossman M , Elman L , McCluskey L , et al. Phosphorylated tau as a candidate biomarker for amyotrophic lateral sclerosis. JAMA Neurol. 2014;71:442‐448. 24492862 10.1001/jamaneurol.2013.6064 PMC3989393

[15] Verde F , Milone I , Colombo E , et al. Phosphorylated tau in plasma could be a biomarker of lower motor neuron impairment in amyotrophic lateral sclerosis. Neurol Sci. 2023;44:3697‐3702. 37369876 10.1007/s10072-023-06916-4

[16] Trautwig AN , Fox EJ , Dammer EB , et al. Network analysis of the cerebrospinal fluid proteome reveals shared and unique differences between sporadic and familial forms of amyotrophic lateral sclerosis. *bioRxiv*. 2024: 2024.2002.2029.582840. 10.1186/s13024-025-00838-9 PMC12082929 40375307

[17] Neumann M , Sampathu DM , Kwong LK , et al. Ubiquitinated TDP‐43 in frontotemporal lobar degeneration and amyotrophic lateral sclerosis. Science. 2006;314:130‐133. 17023659 10.1126/science.1134108

[18] Ren Y , Li S , Chen S , et al. TDP‐43 and phosphorylated TDP‐43 levels in paired plasma and CSF samples in amyotrophic lateral sclerosis. Front Neurol. 2021;12:663637. 34194383 10.3389/fneur.2021.663637 PMC8236522

[19] Hishizawa M , Yamashita H , Akizuki M , Urushitani M , Takahashi R . TDP‐43 levels are higher in platelets from patients with sporadic amyotrophic lateral sclerosis than in healthy controls. Neurochem Int. 2019;124:41‐45. 30578840 10.1016/j.neuint.2018.12.009

[20] Chatterjee M , Ozdemir S , Fritz C , et al. Plasma extracellular vesicle tau and TDP‐43 as diagnostic biomarkers in FTD and ALS. Nat Med. 2024;30:1771‐1783. 38890531 10.1038/s41591-024-02937-4 PMC11186765

[21] Feng W , Beer JC , Hao Q , et al. NULISA: a proteomic liquid biopsy platform with attomolar sensitivity and high multiplexing. Nat Commun. 2023;14:7238. 37945559 10.1038/s41467-023-42834-x PMC10636041

[22] Traxinger K , Kelly C , Johnson BA , Lyles RH , Glass JD . Prognosis and epidemiology of amyotrophic lateral sclerosis: analysis of a clinic population, 1997–2011. Neurol Clin Pract. 2013;3:313‐320. 24195020 10.1212/CPJ.0b013e3182a1b8ab PMC3787117

[23] Khalil M , Pirpamer L , Hofer E , et al. Serum neurofilament light levels in normal aging and their association with morphologic brain changes. Nat Commun. 2020;11:812. 32041951 10.1038/s41467-020-14612-6 PMC7010701

[24] Fournier CN , Gearing M , Upadhyayula SR , Klein M , Glass JD . Head injury does not alter disease progression or neuropathologic outcomes in ALS. Neurology. 2015;84:1788‐1795. 25832660 10.1212/WNL.0000000000001522 PMC4424128

[25] Moszczynski AJ , Strong W , Xu K , McKee A , Brown A , Strong MJ . Pathologic Thr(175) tau phosphorylation in CTE and CTE with ALS. Neurology. 2018;90:e380‐e387. 29298849 10.1212/WNL.0000000000004899 PMC5791789

[26] Glass JD , Fournier CN , Gearing M . Reader response: pathologic Thr(175) tau phosphorylation in CTE and CTE with ALS. Neurology. 2018;91:578‐579. 30224504 10.1212/WNL.0000000000006192

[27] Feneberg E , Gray E , Ansorge O , Talbot K , Turner MR . Towards a TDP‐43‐based biomarker for ALS and FTLD. Mol Neurobiol. 2018;55:7789‐7801. 29460270 10.1007/s12035-018-0947-6 PMC6132775

[28] Jeon GS , Shim YM , Lee DY , et al. Pathological modification of TDP‐43 in amyotrophic lateral sclerosis with SOD1 mutations. Mol Neurobiol. 2019;56:2007‐2021. 29982983 10.1007/s12035-018-1218-2 PMC6394608

[29] Mackenzie IR , Bigio EH , Ince PG , et al. Pathological TDP‐43 distinguishes sporadic amyotrophic lateral sclerosis from amyotrophic lateral sclerosis with SOD1 mutations. Ann Neurol. 2007;61:427‐434. 17469116 10.1002/ana.21147

[30] Pattle SB , O'Shaughnessy J , Kantelberg O , et al. pTDP‐43 aggregates accumulate in non‐central nervous system tissues prior to symptom onset in amyotrophic lateral sclerosis: a case series linking archival surgical biopsies with clinical phenotypic data. J Pathol Clin Res. 2023;9:44‐55. 36226890 10.1002/cjp2.297 PMC9732680

[31] Ashton NJ , Brum WS , Di Molfetta G , et al. Diagnostic accuracy of a plasma phosphorylated tau 217 immunoassay for Alzheimer disease pathology. JAMA Neurol. 2024;81:255‐263. 38252443 10.1001/jamaneurol.2023.5319 PMC10804282

[32] Neumann M , Kwong LK , Lee EB , et al. Phosphorylation of S409/410 of TDP‐43 is a consistent feature in all sporadic and familial forms of TDP‐43 proteinopathies. Acta Neuropathol. 2009;117:137‐149. 19125255 10.1007/s00401-008-0477-9 PMC2693625

