# Supplementary figures and images for: ALS plasma biomarkers reveal neurofilament and pTau correlate with disease onset and progression
Source: Ann Clin Transl Neurol. 2025 Feb 6;12(4):714–23. doi: 10.1002/acn3.70001 (PMC12040516; doi:10.1002/acn3.70001)

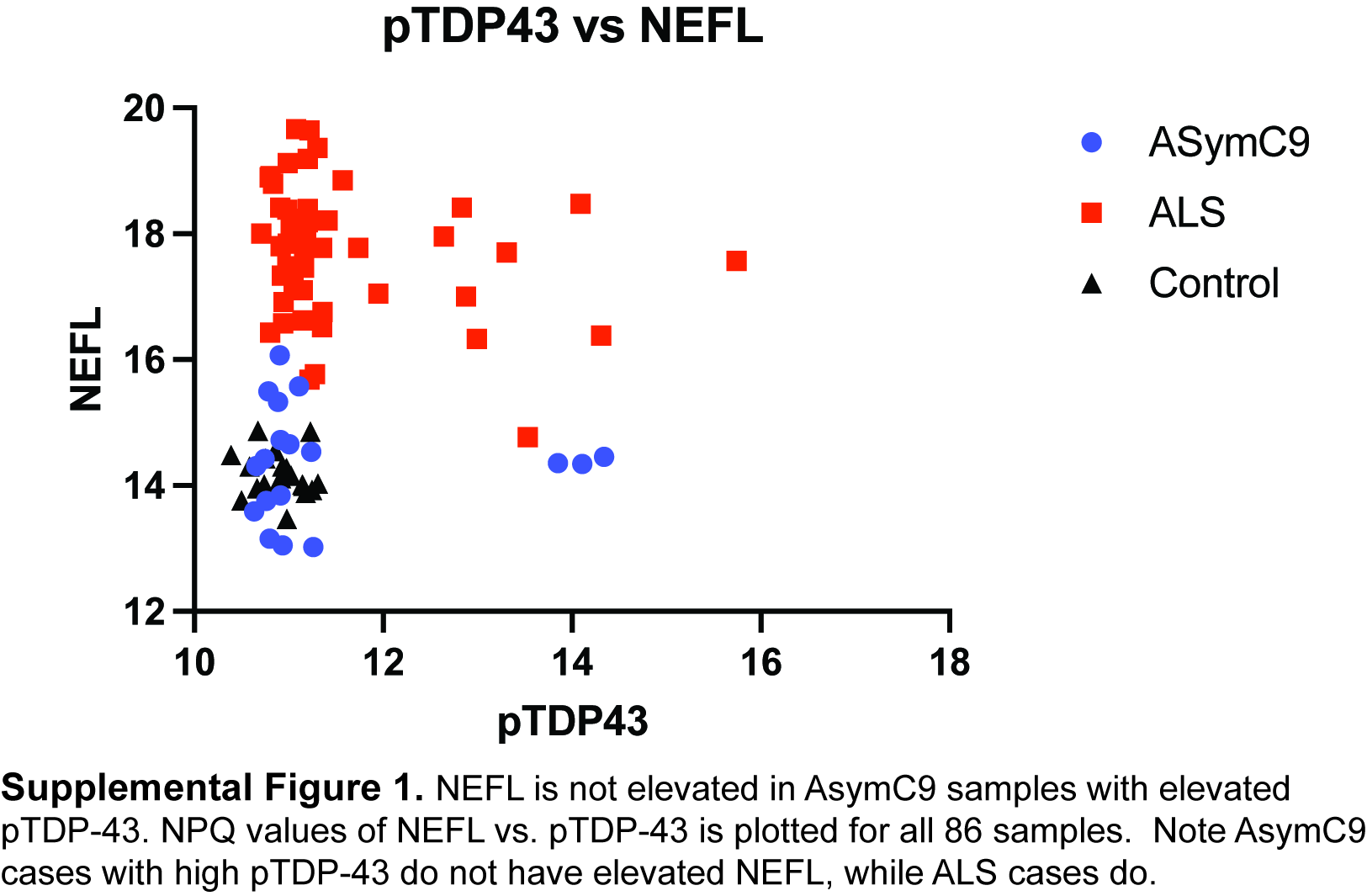

Supplement: Supplementary file 1 — Figure S1. [file ACN3-12-714-s002.tif]

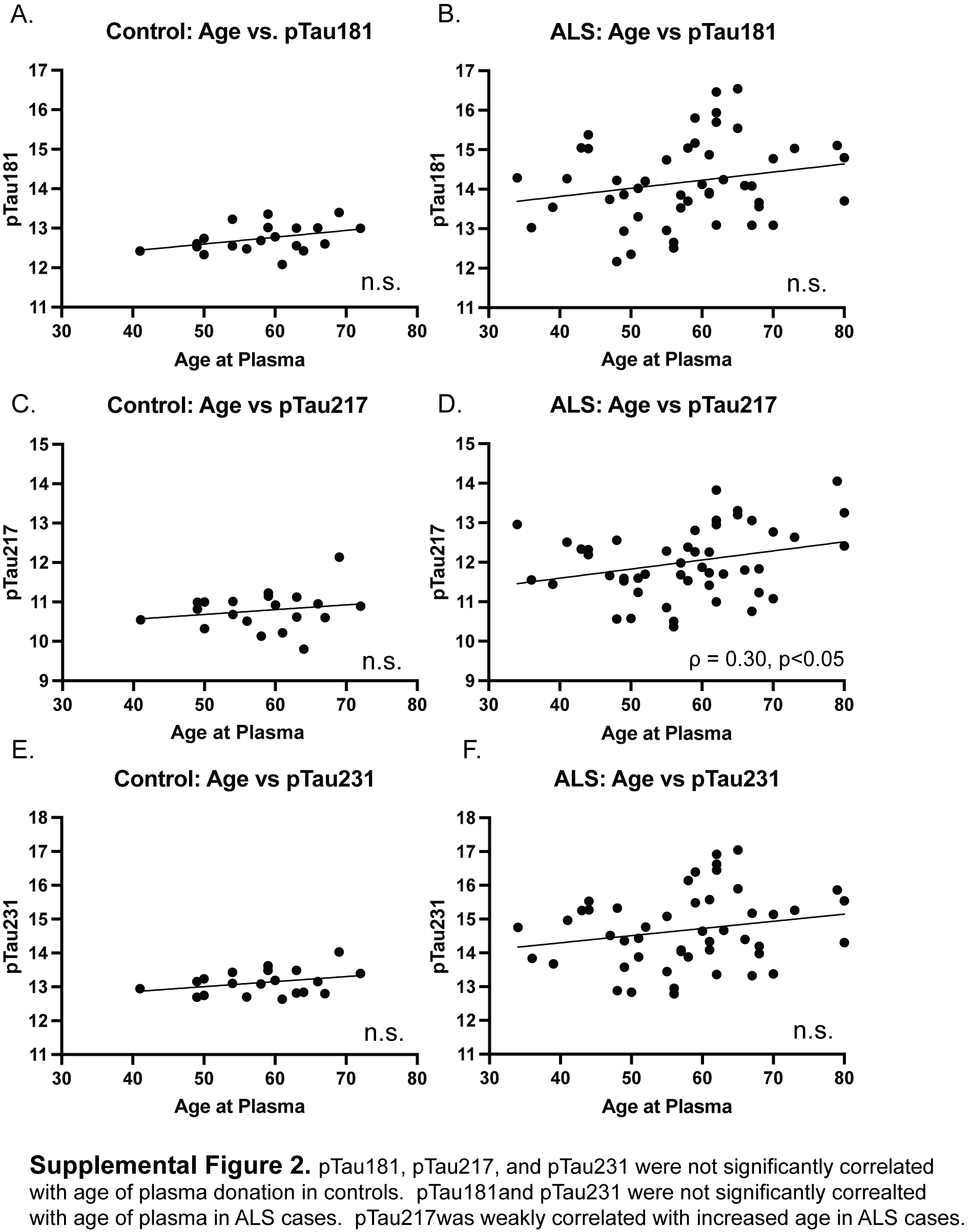

Supplement: Supplementary file 2 — Figure S2. [file ACN3-12-714-s001.tif]
