# Supplementary material for: ALS plasma biomarkers reveal neurofilament and pTau correlate with disease onset and progression
Source: Ann Clin Transl Neurol. 2025 Feb 6;12(4):714–23. doi: 10.1002/acn3.70001 (PMC12040516; doi:10.1002/acn3.70001)
Supplement: Supplementary file 3 — Table S1. [file ACN3-12-714-s004.docx]

**Supplemental Table 1.**

| **Target** | **Protein** | **ANOVA p** |
| --- | --- | --- |
| ACE_38 | Amyloid-beta precursor protein | 1.20E-03 |
| ACE_40 | Amyloid-beta precursor protein | 5.00E-03 |
| ACE_42 | Amyloid-beta precursor protein | 4.50E-02 |
| BACE1 | Beta-site amyloid precursor protein cleaving enzyme 1 | 4.80E-02 |
| CCL4 | C-C motif chemokine ligand 4 | 4.40E-02 |
| CXCL8 | Interleukin 8 |  |
| CX3CL1 | C-X3-C motif chemokine ligand 1 | 9.40E-03 |
| FABP3 | Fatty acid-binding protein 3 | 2.40E-06 |
| GOT1 | Glutamic-oxaloacetic acid transaminase 1 | 5.80E-03 |
| IL10 | Interleukin-10 | 7.70E-03 |
| IL33 | Interleukin-33 | 4.00E-02 |
| MAPT | Microtubule-associated protein tau | 0.00095 |
| NEFH | Neurofilament heavy chain | 4.80E-16 |
| NEFL | Neurofilament light chain | 1.70E-27 |
| NGF | Nerve growth factor | 9.60E-03 |
| POSTN | Periostin | 4.00E-03 |
| PSEN1 | Presenilin-1 | 3.20E-02 |
| pTau181 | phosphorylated tau 181 | 6.30E-11 |
| pTau217 | phosphorylated tau 217 | 1.70E-10 |
| pTau231 | phosphorylated tau 231 | 1.50E-11 |
| pTDP43-409/410 | phosphorylated TDP-43 | 3.60E-02 |
| S100A12 | S100 calcium binding protein A12 | 2.50E-02 |
| UCHL1 | Ubiquitin carboxyl-terminal hydrolase 1 | 0.00017 |
