# Supplementary material for: ALS plasma biomarkers reveal neurofilament and pTau correlate with disease onset and progression
Source: Ann Clin Transl Neurol. 2025 Feb 6;12(4):714–23. doi: 10.1002/acn3.70001 (PMC12040516; doi:10.1002/acn3.70001)
Supplement: Supplementary file 4 — Caption S1. [file ACN3-12-714-s003.docx]

**Supplementary Table 1.**

List of all NULISASeq^TM^ CNS panel analytes with statistically significant (p<0.05) differences in NPQ levels.

**Supplemental Figure 1.**

NEFL is not elevated in AsymC9 samples with elevated pTDP-43. NPQ values of NEFL vs. pTDP-43 is plotted for all 86 samples. Note AsymC9 cases with high pTDP-43 do not have elevated NEFL, while ALS cases do.

**Supplemental Figure 2.**

Phosphorylated tau (pTau181, pTau217, and pTau231) was not significantly correlated with age of plasma donation in controls. pTau181 and pTau231 were not significantly correlated with age of plasma in ALS cases. pTau217 was weakly correlated with increased age of ALS cases.
